# The role of cellular and molecular neuroimmune crosstalk in gut immunity

**DOI:** 10.1038/s41423-023-01054-5

**Published:** 2023-06-19

**Authors:** Daping Yang, Nicole Almanzar, Isaac M. Chiu

**Affiliations:** Department of Immunology, Harvard Medical School, Boston, MA 02115 USA

**Keywords:** Neuroimmunology, Neuroimmune, Host defense, Gut, Sensory, Neuroimmunology, Mucosal immunology

## Abstract

The gastrointestinal tract is densely innervated by the peripheral nervous system and populated by the immune system. These two systems critically coordinate the sensations of and adaptations to dietary, microbial, and damaging stimuli from the external and internal microenvironment during tissue homeostasis and inflammation. The brain receives and integrates ascending sensory signals from the gut and transduces descending signals back to the gut via autonomic neurons. Neurons regulate intestinal immune responses through the action of local axon reflexes or through neuronal circuits via the gut-brain axis. This neuroimmune crosstalk is critical for gut homeostatic maintenance and disease resolution. In this review, we discuss the roles of distinct types of gut-innervating neurons in the modulation of intestinal mucosal immunity. We will focus on the molecular mechanisms governing how different immune cells respond to neural signals in host defense and inflammation. We also discuss the therapeutic potential of strategies targeting neuroimmune crosstalk for intestinal diseases.

## Introduction

While the immune and nervous systems have traditionally been studied separately, it is increasingly clear that these two complex systems are intricately linked at the functional level. Neuronal crosstalk with the immune system is an old concept. Two thousand years ago, the Roman physician Aulus Cornelius Celsus defined the four cardinal signs of inflammation as pain, redness, swelling, and heat; the first sign was driven by the sensory nervous system, and the latter three were linked to vascular and immune functions [1]. Neuronal regulation of host defenses against pathogens is also evolutionarily conserved, with empirical evidence from simple metazoans, including *C. elegans*, to vertebrates, including fish and mammals [2]. The past decades have witnessed major discoveries revealing the pleiotropic roles of neuroimmune crosstalk in physiology, host defense, repair, and pathology [3]. The focus of this review is to discuss advances in this field based on neuroimmune interactions in the gastrointestinal tract and on organ system, where this crosstalk plays a prominent role in homeostasis and disease.

The primary function of the gastrointestinal tract is food processing and digestion. As such, it is densely innervated by peripheral neurons, including sensory and autonomic neurons, that coordinate the detection of nutrients, gut motility, and the secretion of enzymes necessary for proper digestive functions. The GI tract is also populated by a myriad of immunocytes, including both innate and adaptive immune cells, which critically mediate gut tissue homeostasis and host defense against enteric pathogens [4]. Furthermore, the gut is colonized by a community of microbes, including bacteria, viruses, and fungi, that form symbiotic relationships with the host [5]. There is now no doubt that for proper gastrointestinal function and tissue maintenance, neurons must be able to sense stimuli, including those from microbes and immune cells, to mediate both sensory and motor functions in the gut.

**Fig. 1:**
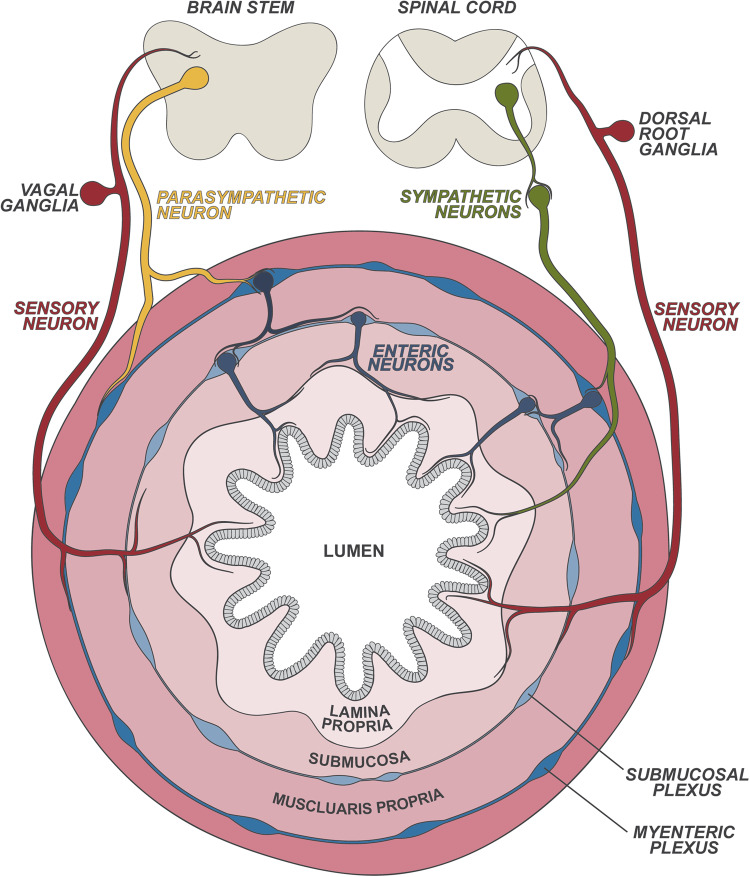
Neuroanatomy of the gastrointestinal tract. The gastrointestinal tract is innervated by gut-extrinsic and gut-intrinsic sensory and autonomic neurons. Extrinsic parasympathetic, sympathetic and sensory neurons originate in the brainstem and spinal cord, projecting to the outer muscle layers and the inner mucosa of the gut. Sensory neurons are pseudounipolar neurons that have their cell bodies in the dorsal root ganglia (DRG) and nodose/jugular vagal ganglia (VG), and they transduce signals from the gut to the spinal cord and brainstem, respectively. Sympathetic neurons consist of preganglionic neurons in the spinal cord that project to the sympathetic ganglia, where they synapse with postganglionic neurons that project to the gut. Parasympathetic neurons communicate from the brainstem to the gut via the vagus nerve. Gut-intrinsic enteric neurons reside in the myenteric and submucosal plexus layers of the intestine and innervate all intestinal layers

The neuroanatomy of the gut is composed of both sensory and autonomic neurons that reside within and outside the organ (Fig. 1). Gut-extrinsic sensory neurons reside in the nodose/jugular vagal ganglia (VG) and the dorsal root ganglia (DRG), taking signals from gut to the brainstem and spinal cord, respectively. Autonomic neurons also innervate the gut, which includes vagal efferent parasympathetic motor neurons and sympathetic neurons that reside in the autonomic ganglia [1, 6]. The gut also houses its own intrinsic and autonomous nervous system, comprised of enteric neurons, with their cell bodies in the myenteric plexus and submucosal plexus [1, 6]. In response to external or internal perturbations, one or more branches of gut-innervating neurons are activated, and neuronal reflexes occur that are essential for their communication with the central nervous system (CNS) and the gut. For example, vagal sensory neurons transduce action potentials to the brainstem, where information is processed, integrated, and perceived, and then descending signals are transmitted via vagal efferent motor neurons back to the gut. Efferent motor neuron activation also results in neurotransmitter release, such as the release of acetylcholine from parasympathetic neurons or catecholamines (norepinephrine, epinephrine) from sympathetic neurons, which can act directly on immune cells to tune their function.

The nervous system and immune system have evolved a common language to communicate with each other at every step of their response to environmental insults from inception to resolution [2]. Neurons express many receptors that are canonically expressed in immune cells, including pattern recognition receptors such as Toll-like receptors (TLRs) and inflammatory cytokine receptors, which allow immune cells to modulate neuronal activity. For example, the inflammatory cytokine IL-1β sensitizes sensory neurons to regulate pain in the context of inflammation [7]. Immune cells are also able to sense neuron-derived cues by virtue of expressing receptors for neurotransmitters and neuropeptides. For example, innate lymphoid cells express receptors for the neuropeptides calcitonin gene-related peptide (CGRP) and neuromedin U (NMU) [8–10]. The mechanism of communication between the nervous and immune systems evolutionarily makes sense, as it decreases the cost of dealing with certain insults and enables the two systems to coordinate complex host responses. The microbiome also plays a critical role in regulating neuronal activation and immune development. Given that both immune cells and neurons can sense microbes directly or indirectly, the composition of the microbiome plays a critical role in neuronal programming or maturation to modulate visceral pain, gut motility, and other aspects of intestinal physiology [11–14].

Here, we focus on mechanisms underlying the neuronal regulation of inflammation and immunity in the gut. We discuss how neurons and neurotransmitters are involved in immune responses during tissue repair and host defense. Advances and challenges coexist in this fascinating field. We highlight the most recent studies that take advantage of new tools to overcome the limitations, and we argue that targeting neurons is a promising strategy for the treatment of diseases in the gut. We are unable to comprehensively cover this topic, as it is a fast-moving field. For further reading on the gut neuro-immune axis and how the microbiome regulates these interactions, we recommend these excellent reviews [15–18].

## Sensory neuron regulation of gut immunity

Gut-innervating sensory neurons arise from the DRG and the nodose/jugular vagal ganglia (VG), transducing signals from the gut to the CNS to mediate the perception of various luminal stimuli, including mechanical stretch, nutrients, microbial cues and immune mediators (Fig. 1). Sensory neurons that are responsible for the unpleasant sensation of pain are called nociceptors. Nociceptor neurons express ion channels such as transient receptor potential (TRP) channels, ATP sensors such as P2X channels, and G-protein coupled receptors (GPCRs) that allow them to sense noxious or harmful stimuli in the gut, such as bacterial pathogens, damaging foods and harmful chemicals. Capsaicin, the active ingredient in chili peppers and spicy food, also activates nociceptor neurons. Therefore, nociceptors play a key role in serving as an alarm system, warning the host of the presence of external and internal insults by mediating nausea, visceral pain, and other protective reflexes [19].

**Fig. 2:**
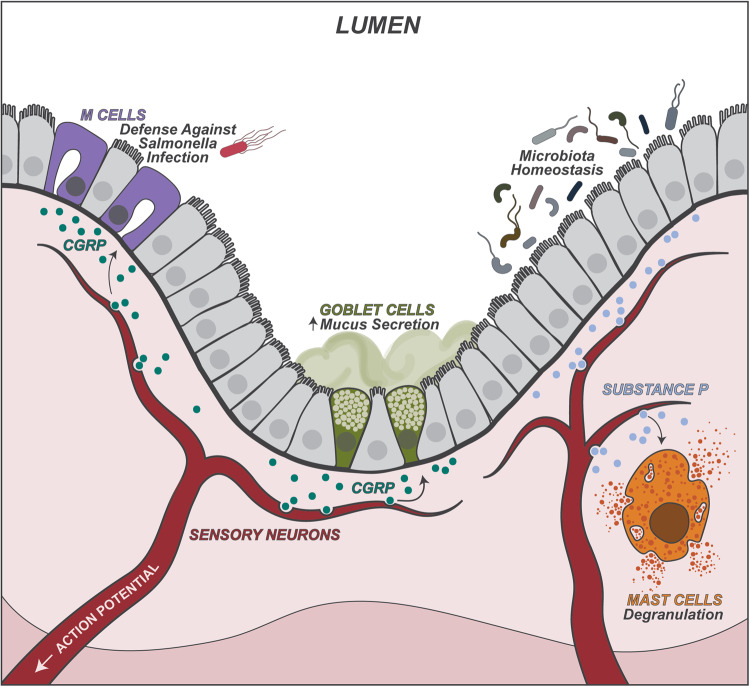
Sensory neuron regulation of gut immunity. In response to microbial and dietary cues, TRPV1^+^Nav1.8^+^ DRG nociceptor neurons are activated, leading to release of the neuropeptides CGRP and Substance P (SP). CGRP suppresses the differentiation of microfold (M) cells in the Peyer’s patch dome, thereby limiting *S. Typhimurium* pathogen invasion. CGRP also promotes goblet cell mucus production through its co-receptor Ramp1, which mediates gut barrier protection against colitis. SP maintains gut microbiota homeostasis, which contributes to mucosal protection. SP also promotes mast cell degranulation, which contributes to visceral pain and inflammation

An important aspect of sensory neurons is their ability to mediate local neurogenic inflammation [20]. In this process, antidromic axon reflexes between peripheral nerve terminals and calcium influx lead to the immediate release of neuropeptides stored in dense-core vesicles of nociceptive neurons into the gut. These neuropeptides include CGRP, substance P (SP), and vasoactive intestinal peptide (VIP), which can act on vascular smooth muscle cells, endothelial cells, epithelial cells and immune cells to modulate inflammation and immunity in the gut (Fig. 2). Therefore, these neurons have a nontraditional “efferent” role in releasing neuropeptides in the gut that directly regulate immune cells.

Nociceptor neurons communicate with immune cells such as mast cells in the gut to regulate pain and inflammation. Mast cell expansion is a hallmark of irritable bowel syndrome, which is usually accompanied by visceral pain. Mast cells are located close to sensory nerves in the gut mucosa, and mediators derived from mast cells, such as histamine, PGE2 and tryptase, are able to enhance the excitation of DRG neurons, leading to visceral pain [21, 22]. One recent study showed that during IgE-mediated anaphylaxis, activated mast cells secrete chymase binding to the receptor protease-activated receptor-1 on Trpv1+ neurons, which modulate the body’s thermoregulatory neural network and cause hypothermia [23]. Sensory neurons can secrete substance P to facilitate mast cell activation, leading to the release of cytokines and chemokines that cause diarrhea, inflammation and altered motility during inflammatory bowel disease pathogenesis [24]. Substance P is also involved in defense responses against *Salmonella* infection, as mice lacking its receptor neurokinin-1 receptor (NK-1R) had enhanced host protection [25]. Whether mast cells contribute to this process needs to be further explored. TRPV1+ vagal neurons that innervate the liver are also capable of detecting nutritional and microbial cues that flow from the gut to the liver via the hepatic portal vein [26]. These vagal afferent neurons transduce signals from the liver to the nucleus tractus solitarius (NTS) of the brainstem, which then relay the signal to the gut via vagal parasympathetic neurons to regulate the differentiation of gut residing regulator T (pTreg) cells [26]. This illustrates that the liver-gut-brain axis senses and regulates gut immunity, which requires autonomic neurons.

Recent studies have shown that DRG neurons play critical roles in gut barrier protection, microbial homeostasis, and protection against colitis [27, 28]. Goblet cells are the intestinal epithelial cells that produce mucus, which coats intestinal surfaces and serves as the first defensive barrier against external dangers. Nav1.8+ nociceptor neurons signal to intestinal goblet cells via the CGRP-Ramp1 signaling axis to regulate mucus production and mediate barrier protection [27]. Nav1.8+ nociceptor neuron ablation in mice leads to decreased colonic mucus layers, accompanied by a dysregulated gut microbiota [27]. These neurons secrete the neuropeptide CGRP in response to luminal stimuli, including microbial cues and capsaicin [27]. Loss of nociceptors leads to increased susceptibility to dextran sulfate sodium (DSS)-induced colitis in mice. Nociceptor activation through chemogenetics or administration of CGRP enhances mucus production and protects mice against colitis pathogenesis [27]. The protective role of CGRP is also consistent with previous studies showing that CGRP-deficient mice are susceptible to colitis [29, 30]. Another independent study also showed that chemogenetic inhibition or pharmacological ablation of Trpv1+ neurons exacerbates DSS-induced colitis through dysregulation of the gut microbiota [28]. Trpv1+ neuron ablation leads to microbial dysbiosis in the mouse colon, and transplantation of the dysregulated microbiota to germ-free mice renders the host susceptible to colitis pathogenesis. Conversely, depletion of the gut microbiota ameliorates colitis pathogenesis in neuron-ablated mice [28]. In this study, substance P (SP), another neuropeptide secreted by nociceptor neurons, mediated host protection against colitis and helped maintain microbiota homeostasis [28]. These two complementary studies indicate that sensory neurons are critical for maintaining mucus production and microbial homeostasis, which protect the gut barrier.

Nociceptor neurons are also critical for host defense against enteric infections. TRPV1+Nav1.8 + DRG neurons protect against infections caused by the gram-negative bacterial pathogen *Salmonella typhimurium* in mice [31]. *S. typhimurium* penetrates the gut barrier through microfold (M) cells, specialized epithelial cells in the small intestine ileum Peyer’s patch (PP) follicle-associated epithelia (FAE) [31]. Nociceptor neurons respond to *S. typhimurium* infection by releasing CGRP, which reduces M cell numbers to limit *S. typhimurium* infection. Nociceptors also regulate the level of the gut commensal microbe segmented filamentous bacteria (SFB), which colonizes the ileum villi and PP FAE to promote host defense against infection [31]. TRPV1+ neurons and other neuropeptides, including SP, VIP, and PACAP, also mediate host defense against pathogenic infections induced by *Citrobacter rodentium* and enterotoxigenic *Escherichia coli* [32].

Sensory neuron activity is also dictated by multiple other cell types in the gut. Enteroendocrine cells (EECs) are specialized gut epithelial cells that form synaptic-like structures with enteric and sensory neurons. In response to microbial products, chemical irritants and dietary stimuli, EECs secrete the neurotransmitter serotonin, which binds to the receptor 5HT_3_R expressed on neurons to modulate afferent mechanosensory functions [33]. EECs also mediate food-induced defensive responses such as nausea and retching through vagal sensory neurons and transmit toxin-induced signals to Tac1+ neurons in the dorsal vagal complex (DVC) of the brainstem; blocking 5HT_3_R signaling blocks toxin-induced nausea-like behaviors [34].

Therefore, sensory neurons play pleiotropic roles in gut barrier protection under both homeostasis and host defense by signaling to immune and epithelial cells (Fig. 2). Remaining questions exist on the detailed cellular and molecular mechanisms that lead to activation of these neurons and their role in protection and host defense against other gut inflammatory triggers such as viral infection, helminth infection, allergic immunity, and autoimmune disease.

## Autonomic neuronal regulation of gut immunity

Autonomic neurons that regulate involuntary physiologic processes in the gut, including digestion and motility, also play a critical role in controlling gut immunity. Autonomic neurons that innervate the gastrointestinal system include enteric neurons, sympathetic neurons, and parasympathetic neurons (Fig. 1). While sympathetic neurons originate from the spinal cord and sympathetic ganglia, parasympathetic neurons originate from the brainstem. Sympathetic neurons mediate the “flight or fight” stress response and execute inhibitory functions, including slowing gut motility and secretion. Parasympathetic neurons mediate excitatory functions, including promotion of gut motility, digestion, and secretion. In contrast, gut-intrinsic enteric neurons have their cell bodies and axons fully within the gut. Enteric neurons are organized into ganglionated networks from the myenteric plexus and submucosal plexus that can be called the “second brain” to regulate gut physiology. Sympathetic and parasympathetic neurons also form synaptic connections with enteric neurons to orchestrate their responses. Each of these distinct neuronal subsets is capable of signaling to immune cells by releasing neurotransmitters into various layers of the gut, which can act on innate and adaptive immune cells.

## Enteric neuron regulation of gut immunity

**Fig. 3:**
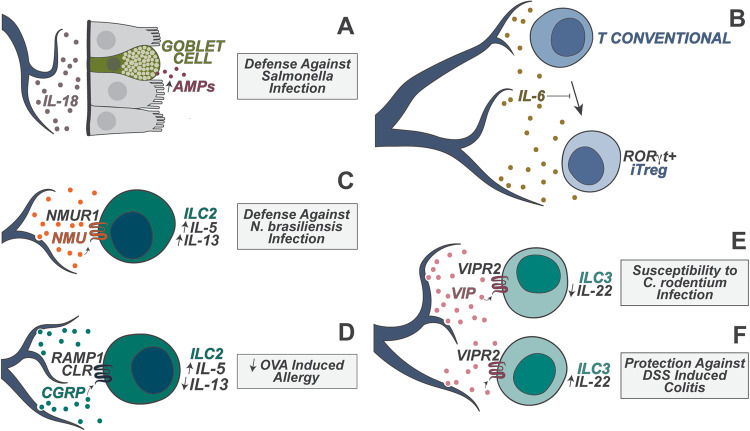
Enteric neuron regulation of gut immunity. Enteric neurons are heterogeneous and can release cytokines (e.g., IL-18 and IL-6) and neuropeptides (e.g., neuromedin U (NMU), calcitonin gene-related peptide (CGRP) and vasoactive intestinal peptide (VIP)) to regulate immune function. **A** Neuronal IL-18 regulates goblet cell expression of antimicrobial peptides, which mediates host protection against intestinal *S. Typhimurium* infection. **B** Neuronal IL-6 inhibits the differentiation of RORγ+ regulatory T cells (iTregs) in the colon. **C** NMU promotes NMUR-dependent ILC2 production of IL-5 and IL-13, which promotes host defense against *N. braliensis* infection. **D** CGRP downregulates IL-13 production by ILC2s, ameliorating OVA-induced allergy. **E** VIP suppresses IL-22 production by ILC3s in a VIPR2-dependent manner, compromising host defense against *C. rodentium* infection. **F** VIP boosts IL-22 production by VIPR2-expressing ILC3s, protecting the host from DSS colitis

Enteric neurons are capable of sensing various gut luminal stimuli and crosstalk between immune cells and epithelial cells in both healthy and disease states via the production of neurotransmitters, neuropeptides, and cytokines (Fig. 3). Enteric neurons in the myenteric plexus crosstalk with resident muscularis macrophages (MMs) to mediate peristalsis, a fundamental aspect of digestion. Enteric neurons sense commensal microbiome-derived cues to secrete CSF1 (mCSF), whose receptor CSF1R is highly expressed on MMs [35]. CSF1R signaling is indispensable for the development of MMs, which in turn produce BMP that acts on enteric neurons to modulate intestinal peristalsis [35]. This bidirectional crosstalk coordinates the contraction and relaxation of smooth muscle cells to regulate intestinal peristalsis in response to microbial and nutritional cues.

Enteric neurons also express cytokines that can regulate both immune and epithelial barrier function as a part of host defense. IL-6, a typical inflammatory cytokine produced by immune cells such as macrophages, is also expressed in enteric neurons [36]. Enteric neuron-derived IL-6 inhibits the differentiation of induced RORγ+ regulatory T cells (iTregs) in the colon. Neuronal-specific IL-6 depletion leads to increased iTreg numbers [36]. The gut microbiome helps shape this enteric neuron-Treg axis, as certain microbes stimulate the loss of enteric neuron networks upon colonization in the gut, which leads to increased iTreg numbers [36]. Enteric neurons express IL-18, which has been found to signal to goblet cells to protect against enteric pathogens. IL-18, which can be produced by immune cells and epithelial cells, is indispensable for antimicrobial peptide (AMP) production, which is required to help maintain microbiota homeostasis and combat pathogen infections [37]. A study showed that enteric neuron-derived IL-18 plays a nonredundant role in regulating AMP production from goblet cells, where enteric neuron-specific IL-18 deficiency results in microbial dysbiosis and susceptibility to *S. typhimurium* infection [38]. It has not been determined how IL-18 is activated and released from enteric neurons.

Enteric neurons also play a major role in coordinating the function of innate lymphoid cells (ILCs) in the gut. ILCs are early responding innate lymphocytes that coordinate downstream adaptive immunity. Enteric neurons are in close proximity to ILCs, laying a cellular basis for these neurons to regulate ILC functions [39, 40]. ILC2s highly express NMUR1, which is the receptor for the neuropeptide neuromedin U (NMU). A subset of enteric sensory neurons express NMU, which is released in allergic conditions. Enteric neurons secrete NMU to boost ILC2 activation and type 2 cytokine production during *N. brasiliensis* helminth infection, leading to improved host protection and worm clearance [39]. Both the alarmin cytokine IL-33 and products from *N. brasiliensis* can trigger NMU production from enteric neuron organoids in a MyD88-dependent manner in vitro, suggesting that these neurons are also responding in a transcriptionally plastic manner to type 2 immune triggers [40]. In contrast to NMU, the neuropeptide CGRP, which is also expressed by enteric neurons and extrinsic sensory neurons, antagonizes ILC2 proliferation and IL-13 expression in helminth infection and intestinal type 2 immunity [8, 41]. ILC2s themselves also upregulate CGRP during infection, which mediates autoinhibition of IL-13 expression [41]. Given the contrasting roles of NMU and CGRP in type 2 immunity host defense, it remains unknown how the release or expression of these neuropeptides are coordinated during infection.

Enteric neurons also play a key role in regulating the function of type 3 ILCs (ILC3s) in the gut lamina propria. ILC3s express high levels of VIPR2, a receptor for the neuropeptide VIP. A subset of enteric neurons (as well as vagal sensory neurons) express high levels of VIP, and they have been found to regulate ILC3 function at homeostasis and during host defense [42, 43]. In one study, food consumption triggered neuronal production of VIP in a manner dependent on circadian rhythms; VIP then inhibited ILC3-mediated production of IL-22 and abrogated intestinal expression of AMPs [42]. Chemogenetic activation of VIP+ neurons results in a decreased proportion of IL-22 + ILC3s and renders the host susceptible to oral *Citrobacter rodentium* infection [42]. A contrasting study showed that VIP promotes ILC3 expansion and IL-22 production, which is abrogated in VIPR2-deficient mice [43]. In this study, VIPR2-deficient mice displayed constitutively fewer IL-22^+^ ILC3s, along with enhanced susceptibility to DSS-induced colitis [43]. Another study showed that VIP is also able to recruit ILC3s into the intestine and that either VIP or VIPR2 deficiency impairs ILC3 recruitment and IL-22 production and renders mice susceptible to *Citrobacter rodentium* infection [44]. Interestingly, one recent study revealed that VIP is able to potentiate both ILC3 and ILC2 activation by synergizing with the cytokines IL-23 or IL-33, thereby boosting ILC-mediated host immunity against *Citrobacter rodentium* or *Trichuris muris* infection, respectively [45]. These studies used somewhat different approaches for the blockade or inhibition of VIP+ neuron activity, but it has not been determined why there were distinct results. Furthermore, the signaling mechanisms within the ILC3s following VIPR2 stimulation that lead to cytokine regulation and transcriptional changes are worthy of future studies.

## Sympathetic neuron regulation of gut immunity

Sympathetic neurons are efferent neurons of the gut-brain axis and the major branch of the stress signaling response. Their main effector neurotransmitters are catecholamines, which bind to adrenergic receptors on target cells. Sympathetic neuron activity is modulated by luminal microbial cues in the gut. Loss of the microbiome leads to increased expression of cFos, a marker of neuronal activation, in sympathetic neurons, suggesting that the endogenous flora plays a regulatory role in suppressing sympathetic neuron activation [46]. Accompanying the increased sympathetic neuron activation, microbiota-depleted mice display slower gut motility, which can be partially rescued by blockade of catecholamine release [46]. Sympathetic nerve fibers spread along the blood vessels in the colon, and local neuron activation also reduces immune cell extravasation into the colon [47]. Endothelial cells express MAdCAM-1, a cell adhesion molecule responsible for leukocyte migration. Optogenetic activation of sympathetic neurons decreases MAdCAM-1 expression on endothelial cells, which mitigates DSS-induced colitis [47]. Blockade of beta-adrenergic receptor signaling reverses the decrease in MAdCAM-1 expression induced by sympathetic neuron activation, suggesting that norepinephrine plays an important role in regulating immune cell migration [47].

In addition to regulating gut motility and immune cell migration, sympathetic neurons modulate intestinal immune cell plasticity. Tyrosine hydroxylase (TH+) sympathetic neurons have close proximity to muscularis macrophages (MMs) in the intestinal muscularis layer. MMs display different morphology and cell dynamics compared with lamina propria macrophages (LpMs), with the former having a tissue protective and wound healing “M2-like” macrophage signature and the latter having an “M1-like” macrophage signature [48]. MMs specifically express the β2 adrenergic receptor, and the main source of norepinephrine that drives MM polarization is gut extrinsic sympathetic neurons [48]. *Salmonella typhimurium* (SpiB mutant) infection activates sympathetic neurons, resulting in NE release in the myenteric plexus; this signals to MMs to polarize their transcriptional profile in vivo in a β2AR-dependent manner [48]. *S. typhimurium* infection also leads to the loss of intrinsic enteric-associated neurons (iEAN), resulting in decreased gut motility [49]. The sympathetic neuron-MM axis helps protect these neurons. It was found that MMs upregulate arginase 1 (Arg1), which helps preserve iEAN cell health during enteric infection through polyamine synthesis [49]. β2AR ablation in MMs exacerbates *S. typhimurium* Spib infection-induced neuronal loss, while sympathetic neuron activation protects the gut from pathogen-induced iEAN damage [49]. This sympathetic neuron-MM mediated neuroprotective axis, connected through MMs intrinsic β2AR signaling, is also important under the setting of repeated infections by multiple enteric pathogens, including *Salmonella*, *Yersinia*, and helminths [50]. In this study, a primary pathogen infection induced β2AR signaling activation in MMs, which protected the gut from subsequent infection by another unrelated pathogen [50].

Sympathetic neurons also signal to ILC2s in the small intestine, where these cells are closely located in the villi and submucosa. Similar to macrophages, ILC2s also highly express the receptor β2AR [51]. β2AR signaling was found to potently inhibit the proliferation of ILCs. β2AR deficiency leads to increased numbers of IL-5^+^ and IL-13^+^ ILC2s and protects the host from gastrointestinal helminth *Nippostrongylus brasiliensis* (*N. brasiliensis*) infection, while β2AR treatment inhibits ILC2-mediated type 2 immune responses and compromises the hosts protection against helminths [51].

**Fig. 4:**
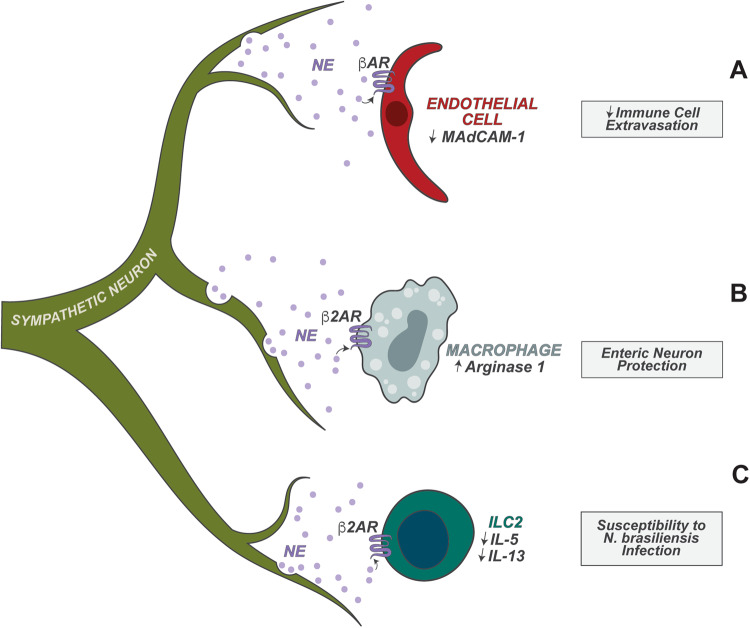
Sympathetic neuron regulation of gut immunity. Sympathetic neuron activation leads to the release of catecholamines, such as norepinephrine (NE), which regulates immunity. **A** NE inhibits the expression of MAdCAM-1 on blood vessel endothelial cells in a βAR-dependent manner, limiting immune cell extravasation during colitis; **B** NE enhances arginase 1 expression and polyamine synthesis in macrophages in a β2AR-dependent manner, preventing enteric neuron cell death; **C** NE limits IL-5 and IL-13 production by β2AR-expressing ILC2s, which weakens host defense against *N. braliensis* infection

These studies together show that sympathetic neurons and local stress signaling through catecholamines can powerfully regulate gut homeostasis and immunity against pathogens (Fig. 4). Allowing for the wide expression of adrenergic receptors on immune cells and endothelial cells, it remains to be determined how these different stress signaling immunoregulations are coordinated in each context.

## Parasympathetic neuron regulation of gut immunity

**Fig. 5:**
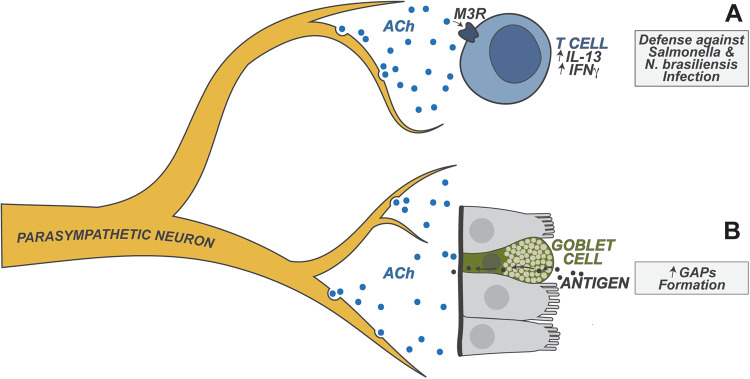
Parasympathetic neuronal regulation of gut immunity. Parasympathetic neurons release the neurotransmitter acetylcholine (Ach), which regulates immunity. **A** Ach enhances T-cell production of IL-13 and IFNγ, facilitating host defense against intestinal Salmonella and *N. braliensis* infections; **B** Ach promotes luminal antigen sampling through goblet cell-associated antigen passages (GAPs), resulting in antigen presentation by tissue CD103+ DCs

Parasympathetic neurons reside in the brainstem and innervate peripheral organs via the vagus nerve. The vagus nerve may directly innervate the gut or signal through intermediary ganglia that in turn innervate the gut. The main neurotransmitter produced by these neurons is acetylcholine (ACh). It is increasingly clear that vagal efferent parasympathetic neurons have major immunomodulatory effects (Fig. 5). The “cholinergic anti-inflammatory reflex” was first discovered as a neural reflex in which the brain modulates systemic inflammation in response to endotoxic shock [52]. After sensing peripheral inflammation, vagal efferents convey signals to choline acetyltransferase (ChAT)-expressing T cells in the spleen, which relay signals to macrophages via Ach. Ach in turn activates a7 nicotinic acetylcholine receptor signaling to suppress the production of TNFα from macrophages [53]. This axis is also relevant to gut inflammation. ChAT^+^ T cells are recruited into the colon during *C. rodentium* infection, and T-cell-specific ChAT deficiency renders the host more susceptible to *C. rodentium* infection, accompanied by increased expression of TNFα, IL1β, and IL6, indicating that T-cell-derived Ach mediates antimicrobial gut defenses [54]. Vagal parasympathetic neurons also modulate gut immunity in a T-cell-independent manner but rather through the enteric nervous system and macrophages [55]. Intestinal alteration is often accompanied by delayed gut motility and proinflammatory cytokine production. Vagal neuron activation reduces the inflammation caused by intestinal manipulation through crosstalk with myenteric enteric neurons, which have close contact with and inhibit MMs that express the a7 nicotinic acetylcholine receptor [55].

Vagal-derived acetylcholine could also regulate T-cell function during adaptive immunity responses to helminth and enteric pathogen infections. The M3 muscarinic Ach receptor (M3R) has been found to mediate host defense against *N. brasiliensis* and *S. typhimurium* intestinal infection. M3R-deficient mice are more susceptible to both infections and display compromised T-cell activation and cytokine release during infections, suggesting a T-cell-dependent role for Ach in protection against pathogens [56]. Ach and M3R agonists promote IL-13 and IFNγ production from T cells in an M3R-dependent manner [56].

Ach also plays a major role in regulating gut epithelial barrier protection. Ach is the most well-known neurotransmitter that regulates goblet cell mucus secretion [57]. Goblet cell-associated antigen passages (GAPs) deliver luminal antigens across the epithelium to the underlying antigen presenting cells (APCs) in the lamina propria, which is also modulated by Ach [58]. Recent studies have shown that goblet cells express muscarinic Ach receptor 4, which senses Ach to promote goblet cell mucus secretion and subsequent GAP formation, and is critical for the induction and maintenance of oral tolerance to luminal antigens [59, 60]. Therefore, parasympathetic neurons are critical for maintaining epithelial barrier integrity and immune homeostasis in the gut.

Of note, one of the challenges in studying how vagal parasympathetic neurons regulate gut immunity is the lack of more specific tools to target only these neurons in the brainstem, as these motor efferents are molecularly diverse. It is also difficult to study because cholinergic neurons reside in both the brainstem and the enteric nervous system. Future work will require investigating the role of distinct vagal efferent subsets in regulating gut immunity.

## Brain-to-gut regulation of immunity

A major question in this field is how the brain senses inflammation and crosstalks with the gut from a top-down perspective. Recent work suggests that there could be specific brain regions that respond to colitis and that specific neural circuits provide feedback to the gut to regulate inflammation. Using cFOS-TRAP-based techniques, a recent study showed that intestinal inflammation induced by DSS treatment leads to neuron activation in the thalamus, paraventricular hypothalamic (PVH) nuclei, central amygdala (CeA), anterior cingulate cortex (ACC), supplementary somatosensory cortex, and insular cortex [61]. Chemogenetic reactivation of colitis-responsive insular cortex neurons leads to both leukocyte recruitment and activation in the colon, suggesting that the gut immune status or signature can be retrieved by neuron reactivation in the brain [61]. Conversely, inhibition of these neurons ameliorates DSS-induced inflammation and colitis in the colon [61], suggesting a potential avenue of targeting brain neurons for immune intervention in the gut. The mechanism and neural circuits that connect the insular cortex to the gut to regulate the gut immune response need to be further explored. How neuronal activation in other brain regions affects peripheral immune states in the gut is still relatively unknown. An elegant study related to the spleen showed that neurons in the central nucleus of the amygdala (CeA) and the paraventricular nucleus (PVN) are connected to the splenic nerve, facilitating plasma cell differentiation in the spleen [62]. It would be interesting if similar pathways connected these regions to the GI tract.

Another way the brain affects gut immunity is via neuroendocrine signals, in particular, the hypothalamus pituitary adrenal gland (HPA) axis, which can be activated under conditions of stress and inflammation [63]. In this case, hypothalamic neurons secrete corticotropin releasing factor (CRF), which induces ACTH release from the pituitary gland, and this induces the adrenal gland to produce immunomodulatory hormones, including catecholamines and cortisol, which can circulate to the gut to induce a feedback loop. Stress signaling can also be activated by gut microbiota changes. Gut microbiome deprivation is associated with social deficits and increased release of the stress hormone corticosterone; blocking HPA axis activation corrects social deficits induced by microbiota depletion [64]. In the context of vascular disease, stress-induced HPA axis activation promotes glucocorticoid hormone release, which causes enhanced gut permeability accompanied by increased IL-17 production from Th17 cells [65]. IL-17 in turn amplifies stress-induced inflammation by promoting neutrophil expansion [65]. In the context of a chronic stress model induced by social defeat in mice, intestinal dysbiosis specifically leads to increased expansion of colonic dectin-1+ γδ T cells, which enhances the differentiation and accumulation of IL-17-producing γδ T cells in the colon and meninges and concomitantly results in social avoidance [66]. These studies demonstrate the critical role of stress-induced IL-17 production in mediating pathological and psychological outcomes.

Neurological disorders, including autism spectrum disorder (ASD), have been shown to be accompanied by GI dysfunction [67], suggesting an important role for the brain in regulating gut immunity. The mouse maternal immune activation (MIA) model is commonly used to mimic ASD. MIA offspring that display ASD-like behavioral impairment have deficient gut barrier functions associated with abnormal expression of tight junction molecules and intestinal dysbiosis [67]. However, the cellular basis underlying this gut barrier regulation by the brain is unclear. It would be interesting to determine in situations such as ASD whether dysregulated brain to gut neural circuits regulate gut immunity and barrier function.

Therefore, future work is needed to determine how brain activity and signals can be transduced to the GI tract to affect immunity. It also remains to be determined whether GI dysfunction is the result or cause of neurological disorders.

## Future directions and outlook

Neuroimmunology is a fast-growing field. Recent breakthroughs in how different neuronal subsets regulate immune responses in the gut deepen our understanding of intestinal immunity under physiological and pathological conditions. In addition to passively accepting and responding to harmful insults, the immune system signals to the nervous system to initiate defensive responses. Meanwhile, under the anticipation and perception of potential threats, the nervous system actively modulates the immune response in the gut. Coordination between the nervous system and immune system allows the host to properly deal with complex stimuli and an ever-changing environment. The cellular neural basis of how distinct neuron types regulate gut immunity and the molecular mechanism of how different neurotransmitters modulate immune cells are major questions that need to be characterized in the future.

An important consideration is that neurons and immune cells integrate signals from a complex tissue microenvironment. Therefore, analysis of neuroimmune signaling will require analysis of other cell types. Enteric glial cells, which support enteric neuron development, exhibit immunoregulatory functions by signaling to both immune cells and epithelial cells in the gut [68–70]. Endothelial cells, fibroblasts, and other mesenchymal cells support intestine structure and function, which may also mediate neuroimmune crosstalk. Spatial transcriptomics is one approach that can provide an unbiased picture of cellular composition in the tissue at the transcriptional level [71], and utilizing this approach in neuroimmunology could allow a better picture of how the heterogeneous cellular atlas is regulated by neurons in the gut under physiological and disease conditions.

The gut microbiota is also a critical arm that regulates both neuronal and immune activation in the gut-brain axis. The used of gnotobiotic mice with bacterial genetic manipulation remains the most powerful approach in uncoupling microbiota from the gut-brain axis. Multi-omic studies combining the transcriptome, proteome, metagenome and microbiome will improve our understanding of how neurons shape the intestinal ecosystem [72].

Recent technical advancements have promoted the identification and manipulation of neurons in regulating gut immunity. Optogenetics and chemogenetics have allowed for the manipulation of neuronal activity in a temporally and spatially controlled manner [47, 73, 74], providing an elegant approach to dissect the role of neuronal types in gut immunity. Single-cell sequencing of neurons shows that gut-innervating neurons have diverse cellular compositions within each tissue layer and stimulus modality [75–77]. Studies using neuronal tracing from the gut to the brain and other organs combined with imaging are starting to identify the neural circuits involved in neuro-immune communication. Future research will require targeting specific neuron subsets to elucidate their roles in regulating the intestinal immune response, which will also be the cellular basis of potential therapeutic interventions.

The immunoregulatory role of the brain in gut immunity is an open area of exploration. This includes mapping how emotional and cognitive brain regions could signal to the body to induce intestinal dysfunction. The communication between the brain and gut is bidirectionally mediated by neural, endocrine, immune, and humoral links [78]. One recent study describes the phenomenon that neuron activation in the insular cortex activates colitis-related immune responses in the gut, but the cellular and molecular basis for this regulation still needs to be determined. A comprehensive study dissecting the contribution of distinct factors involved in immune regulation from the brain to the gut under different disease contexts will facilitate our understanding of the gut-brain axis.

These findings also have therapeutic implications for targeting neuroimmune interactions for inflammatory disease treatment. Since the discovery that electrical vagal efferent neuron stimulation attenuates endotoxin-induced inflammation in vivo [52], bioelectronic devices have been used to treat or dampen immune responses in patients with kidney injury, lung injury, and spinal cord injury [79]. The vagus nerve also plays a counter-inflammatory role during colitis in a macrophage-dependent manner by inhibiting proinflammatory cytokine expression while stimulating anti-inflammatory cytokine production, indicating that activating vagal neurons could be a potential strategy for inflammatory bowel disease (IBD) treatment [80–82]. Clinical trials have been undertaken to evaluate the therapeutic effects of noninvasive vagus nerve stimulation on the treatment of Crohn’s disease. Therapeutic targeting of neurotransmitters and neuropeptide receptor signaling could also be another approach to apply neuroimmune principles to treat disease. Medications such as beta-adrenergic receptor antagonists (beta-blockers) developed to treat hypertension, angina pectoris, and cardiac arrhythmias [83] or CGRP receptor antagonists used to treat migraine [84] can be repurposed for gastrointestinal dysfunction interference by virtue of their ability to modulate gut immunity. We also expect to see more translational studies targeting neurons as therapeutic strategies with the development of noninvasive tools such as engineered adenovirus and electroacupuncture [85–87].

The advances and breakthroughs in neuroimmune research in the gut during the last several years have demonstrated the potential of therapeutic strategies that manipulate neuroimmune interactions for clinical disease treatment. There are still challenges that need to be addressed before we can understand this interaction. The neuronal connections and neurotransmitters that mediate neuroimmune crosstalk need to be further characterized, and the role of brain networks in the regulation of peripheral immunity remains elusive. These are the fundamental questions in this realm that we expect to witness more breakthroughs in relation to in the coming years.
